# Supplementary material for: Psychological Burden during the COVID-19 Pandemic in Female Caregivers of Preterm versus Term Born Children
Source: Children (Basel). 2023 Apr 27;10(5):787. doi: 10.3390/children10050787 (PMC10217047; doi:10.3390/children10050787)
Supplement: Supplementary file 1 [file children-10-00787-s001.zip › children-2306057-supplementary.pdf]

Supplementary Materials: The following supporting information can be downloaded at: https://www.mdpi.com/article/10.3390/children10050787/s1,

Assessment of child protection behavior:
The following 11 items were asked to assess child protection behavior.

Table S1. Corrected item-scale correction values of the scale and changes in Cronbach's Alpha.

Scale PB	Corrected Item-Scale Correlation	Changes in Chronbach's Alpha due Item Exclusion
1. I was worried of my child´s health because of COVID-19.	0.455	0.703
2. I washed the hands of my child more often and disinfected contact surfaces more frequently.	0.474	0.700
3. My child wore a breathing mask.	0.267	0.733
4. I thought more about the nutrition of my child.	0.445	0.706
5. I gave my child dietary supplements.	0.323	0.721
6. My child got alternative medication.	0.291	0.725
7. My child went to school by private vehicles.	0.399	0.711
8. I missed social and familiar support because of COVID-19.	0.321	0.723
9. I was worried to take my child to therapies like physiotherapy, ergotherapy or others.	0.535	0.691
10. My child took part in less therapies because of COVID-19.	0.456	0.703
11. I was worried of too less therapies for my child.	0.241	0.733

Note. N = 2382. Scale Cronbach´s alpha = 0.733. A 7-point Likert scale was used for each item (ranging from "1 = strongly disagree" to "7 = strongly agree").

Table S2. Results of descriptive subgroup analysis—prevalence of generalized anxiety symptoms, depression symptoms, COVID-19-anxiety and child protection behavior.

			Medium	Cut-Off Values [%]
Female caregiver´ age	18–24	Generalized anxiety (GAD-7)	6.7	GAD ≥ 5 39.5<br>GAD ≥ 10 16.1<br>GAD ≥ 15 8.1
		Depression (PHQ-2)	2.2	PHQ ≥ 3 33.9
		PB	29.3	
		C-19-A.	19.5	
	25–34	Generalized anxiety (GAD-7)	6.5	GAD ≥ 5 34.5<br>GAD ≥ 10 14.7<br>GAD ≥ 15 8.6
		Depression (PHQ-2)	1.9	PHQ ≥ 3 27.5
		PB	30.4	
		C-19-A.	18.6	
	35–44	Generalized anxiety (GAD-7)	6.7	GAD ≥ 5 33.8<br>GAD ≥ 10 15<br>GAD ≥ 15 9.6
		Depression (PHQ-2)	1.7	PHQ ≥ 3 22
		PB	31.8	
		C-19-A.	19.1	

	45–54	Generalized anxiety (GAD-7)	8.1	GAD ≥ 5 31<br>GAD ≥ 10 22.4<br>GAD ≥ 15 13.8
		Depression (PHQ-2)	1.9	PHQ ≥ 3 25.9
		PB	32.7	
		C-19-A.	20.9	
Marital status	Single	Generalized anxiety (GAD-7)	6.6	GAD ≥ 5 32.3<br>GAD ≥ 10 13.1<br>GAD ≥ 15 10.9
		Depression (PHQ-2)	1.9	PHQ ≥ 3 28.4
		PB	31.1	
		C-19-A.	18.5	
	Married	Generalized anxiety (GAD-7)	6.5	GAD ≥ 5 34.4<br>GAD ≥ 10 15.5<br>GAD ≥ 15 8.5
		Depression (PHQ-2)	1.8	PHQ ≥ 3 24.9
		PB	30.7	
		C-19-A.	18.9	
	Relation-ship	Generalized anxiety (GAD-7)	6.4	GAD ≥ 5 37.6<br>GAD ≥ 10 12.7<br>GAD ≥ 15 8.6
		Depression (PHQ-2)	2.0	PHQ ≥ 3 30.3
		PB	30.6	
		C-19-A.	18.5	
	Divorced	Generalized anxiety (GAD-7)	8.3	GAD ≥ 5 21.5<br>GAD ≥ 10 23.1<br>GAD ≥ 15 16.9
		Depression (PHQ-2)	2.0	PHQ ≥ 3 29.2
		PB	33.6	
		C-19-A.	19.7	
	Widowed	Generalized anxiety (GAD-7)	10.3	GAD ≥ 5 33.3<br>GAD ≥ 10 66.7<br>GAD ≥ 15 0.0
		Depression (PHQ-2)	1.3	PHQ ≥ 3 0.0
		PB	44.0	
		C-19-A.	21.7	
Family Role	Mother	Generalized anxiety (GAD-7)	6.6	GAD ≥ 5 34.6<br>GAD ≥ 10 15.0<br>GAD ≥ 15 9.0
		Depression (PHQ-2)	1.8	PHQ ≥ 3 26.4
		PB	30.8	
		C-19-A.	18.9	
	Foster mother	Generalized anxiety	4.4	GAD ≥ 5 0.0

		(GAD-7)		GAD ≥ 10 20.0
				GAD ≥ 15 0.0
		Depression (PHQ-2)	1.6	PHQ ≥ 3 20.0
		PB	33.6	
		C-19-A.	16.2	
	University	Generalized anxiety (GAD-7)	6.0	GAD ≥ 5 35.1
				GAD ≥ 10 13.3
				GAD ≥ 15 6.4
		Depression (PHQ-2)	1.6	PHQ ≥ 3 21.4
		PB	30.1	
		C-19-A.	18.3	
	Higher educa-tion entrance qualify-cation	Generalized anxiety (GAD-7)	6.7	GAD ≥ 5 34.7
				GAD ≥ 10 14.3
				GAD ≥ 15 10.2
		Depression (PHQ-2)	1.9	PHQ ≥ 3 26.9
		PB	30.6	
		C-19-A.	18.9	
Education level	Secondary ed-ucation	Generalized anxiety (GAD-7)	6.9	GAD ≥ 5 34.1
				GAD ≥ 10 17.8
				GAD ≥ 15 9.6
		Depression (PHQ-2)	2.0	PHQ ≥ 3 30.8
		PB	31.6	
		C-19-A.	19.3	
	Lower second-ary education	Generalized anxiety (GAD-7)	7.3	GAD ≥ 5 27.7
				GAD ≥ 10 15.8
				GAD ≥ 15 14.8
		Depression (PHQ-2)	2.0	PHQ ≥ 3 29.6
		PB	32.4	
		C-19-A.	19.7	
	No qualifica-tion	Generalized anxiety (GAD-7)	9.2	GAD ≥ 5 80.0
				GAD ≥ 10 0.0
				GAD ≥ 15 20.0
		Depression (PHQ-2)	3.2	PHQ ≥ 3 40.0
		PB	28.0	
		C-19-A.	20.0	
Previously diag-nosed psychiatric dis-order	Yes	Generalized anxiety (GAD-7)	10.6	GAD ≥ 5 34.9
				GAD ≥ 10 14.2
				GAD ≥ 15 7.4
		Depression (PHQ-2)	3.0	PHQ ≥ 3 24.4
		PB	32.1	
		C-19-A.	21.4	
	No	Generalized anxiety	6.3	GAD ≥ 5 29.5

		(GAD-7)		GAD ≥ 10 24.8<br>GAD ≥ 15 29.5
		Depression (PHQ-2)	1.7	PHQ ≥ 3 50.9
		PB	30.7	
		C-19-A.	18.6	
		Generalized anxiety (GAD-7)	6.7	GAD ≥ 5 32.9<br>GAD ≥ 10 15.2<br>GAD ≥ 15 10.0
	≥100,000	Depression (PHQ-2)	1.8	PHQ ≥ 3 25.4
		PB	30.7	
		C-19-A.	18.8	
		Generalized anxiety (GAD-7)	6.7	GAD ≥ 5 35.5<br>GAD ≥ 10 15.8<br>GAD ≥ 15 9.2
	≥20,000	Depression (PHQ-2)	1.9	PHQ ≥ 3 28.7
		PB	30.7	
		C-19-A.	18.9	
City size population		Generalized anxiety (GAD-7)	6.4	GAD ≥ 5 34.9<br>GAD ≥ 10 15.6<br>GAD ≥ 15 7.3
	≥5000	Depression (PHQ-2)	1.8	PHQ ≥ 3 24.4
		PB	30.6	
		C-19-A.	18.9	
		Generalized anxiety (GAD-7)	6.4	GAD ≥ 5 35.2<br>GAD ≥ 10 13.8<br>GAD ≥ 15 8.6
	<5000	Depression (PHQ-2)	1.8	PHQ ≥ 3 26.4
		PB	31.1	
		C-19-A.	18.7	
		Generalized anxiety (GAD-7)	6.5	GAD ≥ 5 34.9<br>GAD ≥ 10 14.7<br>GAD ≥ 15 8.4
	Employed	Depression (PHQ-2)	1.8	PHQ ≥ 3 24.4
		PB	30.9	
		C-19-A.	18.7	
Occupation		Generalized anxiety (GAD-7)	7.0	GAD ≥ 5 33.2<br>GAD ≥ 10 16.3<br>GAD ≥ 15 10.9
	Not employed	Depression (PHQ-2)	2.1	PHQ ≥ 3 32.8
		PB	30.5	
		C-19-A.	19.5	
Child´s age	0–3	Generalized anxiety (GAD-7)	6.2	GAD ≥ 5 35.1<br>GAD ≥ 10 13.4<br>GAD ≥ 15 7.6

		Depression (PHQ-2)	1.8	PHQ ≥ 3 24.9
		PB	30.1	
		C-19-A.	18.6	
	4–6	Generalized anxiety (GAD-7)	7.7	GAD ≥ 5 34.5<br>GAD ≥ 10 19.3<br>GAD ≥ 15 12.4
		Depression (PHQ-2)	2.1	PHQ ≥ 3 32.6
		PB	32.4	
		C-19-A.	19.6	
	7–10	Generalized anxiety (GAD-7)	7.4	GAD ≥ 5 32.1<br>GAD ≥ 10 21.0<br>GAD ≥ 15 11.1
		Depression (PHQ-2)	1.8	PHQ ≥ 3 25.8
		PB	32.9	
		C-19-A.	19.2	
	11–17	Generalized anxiety (GAD-7)	8.0	GAD ≥ 5 29.1<br>GAD ≥ 10 16.3<br>GAD ≥ 15 19.8
		Depression (PHQ-2)	2.1	PHQ ≥ 3 31.4
		PB	34.0	
		C-19-A.	20.5	
	32–36 weeks	Generalized anxiety (GAD-7)	6.6	GAD ≥ 5 33.9<br>GAD ≥ 10 13.7<br>GAD ≥ 15 10.1
		Depression (PHQ-2)	1.8	PHQ ≥ 3 21.7
		PB	31.1	
		C-19-A.	19.0	
Gestational age of preterm children	28–32 weeks	Generalized anxiety (GAD-7)	6.4	GAD ≥ 5 32.3<br>GAD ≥ 10 15.1<br>GAD ≥ 15 9.2
		Depression (PHQ-2)	1.7	PHQ ≥ 3 24.9
		PB	32.6	
		C-19-A.	18.8	
	≤28 weeks	Generalized anxiety (GAD-7)	6.0	GAD ≥ 5 29.2<br>GAD ≥ 10 13.5<br>GAD ≥ 15 9.0
		Depression (PHQ-2)	1.4	PHQ ≥ 3 18.0
		PB	32.1	
		C-19-A.	18.5	
Child´s disease: BPD	Yes	Generalized anxiety (GAD-7)	6.9	GAD ≥ 5 27.3<br>GAD ≥ 10 20.0<br>GAD ≥ 15 10.9

		Depression (PHQ-2)	1.6	PHQ ≥ 3 21.8
		PB	35.2	
		C-19-A.	19.9	
	No	Generalized anxiety (GAD-7)	6.4	GAD ≥ 5 33.3<br>GAD ≥ 10 13.4<br>GAD ≥ 15 9.6
		Depression (PHQ-2)	1.7	PHQ ≥ 3 22.1
		PB	31.3	
		C-19-A.	18.7	
Asthma	Yes	Generalized anxiety (GAD-7)	8.4	GAD ≥ 5 28.4<br>GAD ≥ 10 17.6<br>GAD ≥ 15 21.6
		Depression (PHQ-2)	2.3	PHQ ≥ 3 40.5
		PB	34.0	
		C-19-A.	21.2	
	No	Generalized anxiety (GAD-7)	6.5	GAD ≥ 5 34.7<br>GAD ≥ 10 14.9<br>GAD ≥ 15 8.6
		Depression (PHQ-2)	1.8	PHQ ≥ 3 25.9
		PB	30.7	
		C-19-A.	18.8	
Operation	Yes	Generalized anxiety (GAD-7)	6.8	GAD ≥ 5 35.9<br>GAD ≥ 10 16.8<br>GAD ≥ 15 9.9
		Depression (PHQ-2)	1.7	PHQ ≥ 3 24.4
		PB	33.1	
		C-19-A.	19.3	
	No	Generalized anxiety (GAD-7)	6.6	GAD ≥ 5 34.4<br>GAD ≥ 10 15.0<br>GAD ≥ 15 8.9
		Depression (PHQ-2)	1.8	PHQ ≥ 3 26.4
		PB	30.7	
		C-19-A.	18.8	

GAD-7 = generalized anxiety disorder-7, PHQ-2 = patient health questionnaire-2, PB = child protection behavior, C-19-A = COVID-19 anxiety questionnaire.
